# Intraoperative oxygen challenge for toleration of single lung ventilation in a patient with severe obstructive airway disease: A case report

**DOI:** 10.1016/j.amsu.2019.10.032

**Published:** 2019-11-09

**Authors:** Laurence Weinberg, Luka Cosic, Maleck Louis, Tom Garry, Patryck Lloyd-Donald, Stephen Barnett, Lachlan F. Miles

**Affiliations:** a Department of Anaesthesia, Austin Hospital, 145 Studley Road, Victoria, 3084, Australia; b Department of Cardiothoracic Surgery, Austin Hospital, 145 Studley Road, Victoria, 3084, Australia

**Keywords:** Case report, Risk stratification, Anaesthesia, Thoracic surgery, Bronchial blocker, COPD, chronic obstructive pulmonary disease, CPET, cardiopulmonary exercise testing, CT, computed tomography, FEV1, forced expiratory volume in 1 second, FVC, forced vital capacity, TLCO, carbon monoxide transfer factor, SABR, stereotactic ablative radiotherapy, SPECT, single photon emission computed tomography, VE/VCO_2_, minute ventilation/carbon dioxide, VO_2_, maximum oxygen consumption

## Abstract

Perioperative risk assessment is complex in patients with chronic obstructive pulmonary disease who have undergone previous lung resection surgery. A 70-year-old female with severe chronic obstructive pulmonary disease and previous right middle and lower lobectomy, presented for left lower lobe superior segmentectomy. Respiratory function tests revealed a forced expiratory volume in 1 second of 0.72L, a forced vital capacity of 1.93L, and a carbon monoxide transfer factor of 10.0 ml/min/mmHg. A cardiopulmonary exercise test demonstrated little ventilatory reserve with profound arterial desaturation on peak exercise, however, a normal peak oxygen consumption (16.7 ml/min/kg) and a nadir minute ventilation/carbon dioxide slope of 24 implied a limited risk of perioperative cardiovascular morbidity. Given these conflicting results we performed an intraoperative oxygen challenge test under general anaesthesia with sequential ventilation of different lobes of the lung. We demonstrate the use of the oxygen challenge test as an effective intervention to further assess safety and tolerance of anaesthesia of patients with limited respiratory reserve being assessed for further complex redo lung resection surgery. Further, this test was a risk stratification tool that allowed informed decisions to be made by the patient about therapeutic options for treating their lung cancer. The prognostic value of traditional physiological parameters in patients with chronic obstructive pulmonary disease who have undergone previous lung resection surgery is uncertain. The intraoperative oxygen challenge test is another risk stratification tool to assist clinicians in assessment of safety and tolerance of anaesthesia for patients being considered for lung resection.

## Highlights

• Perioperative risk assessment is complex in patients with COPD and previous lung resection.
• Cardiopulmonary exercise testing and pulmonary function tests can be inconclusive for perioperative risk stratification.
• Elective oxygen challenge test is an additional effective risk stratification tool.
• Oxygen challenge test can guide optimum device choice for lung separation.
• Joint shared decision-making processes are predicated upon effective communication of perioperative risk.

## 1 Introduction

Lung resection surgery is the standard curative treatment for lung cancer but is only feasible in patients with local tumour and some preservation of respiratory function [[1], [2], [3], [4], [5]]. The perioperative risk assessment of patients with local tumour and chronic obstructive pulmonary disease (COPD) is challenging. In such patients, the additional use of cardiopulmonary exercise testing (CPET) has been used for risk stratification to further define perioperative risk of morbidity and mortality [[6], [7], [8], [9]]. Perioperative risk assessment is even more complex in patients with severe COPD who have undergone previous chemotherapy and lung resection surgery, as surgery itself leads to a 13%–28% decrease in peak exercise capacity, lasting up to 24 months after resection [10]. We present such a case where the traditional physiological parameters obtained during CPET and pulmonary function tests were unable to provide an informed decision to the patient about the appropriate therapeutic approach to treating their cancer. In this report we demonstrate the use of an oxygen challenge test as an effective intervention to further assess safety and tolerance of anaesthesia of patients with limited respiratory reserve being assessed for further complex redo lung resection surgery. We outline a risk stratification tool to assist in assessment of safety and tolerance of anaesthesia in a patient with severe COPD who had undergone previous lung resection surgery. This case is reported in line with the SCARE criteria [11].

## 2 Presentation of case

A 70-year-old Caucasian female (weight 55kg, height 156cm) was referred for further assessment to a university teaching hospital with a left lower superior segment lesion of the lung. Three years prior she had undergone a right middle and lower lobectomy for a T_2_N_0_M_0_ basaloid squamous cell lung carcinoma. Past medical history was significant for COPD secondary to a 50-pack-year smoking history. Other comorbidities included osteopaenia, stable schizophrenia and depression. There was no history of alcohol use, and the patient was independent for all activities of daily living. The Edmonton Frail Scale score was 8 (mild frailty). Medications included atrovent solution (250 μg/mL inhaled via a nebuliser three times per day), tiotropium bromide capsules (18 mcg daily), ventolin nebules (5 mg inhaled via a nebuliser three times daily), combination budesonide and fumoterol fumarate dihydrate inhaler (two puffs twice daily), vitamin D (1000 IU daily) and quetiapine (300mg daily).

**Fig. 1:**
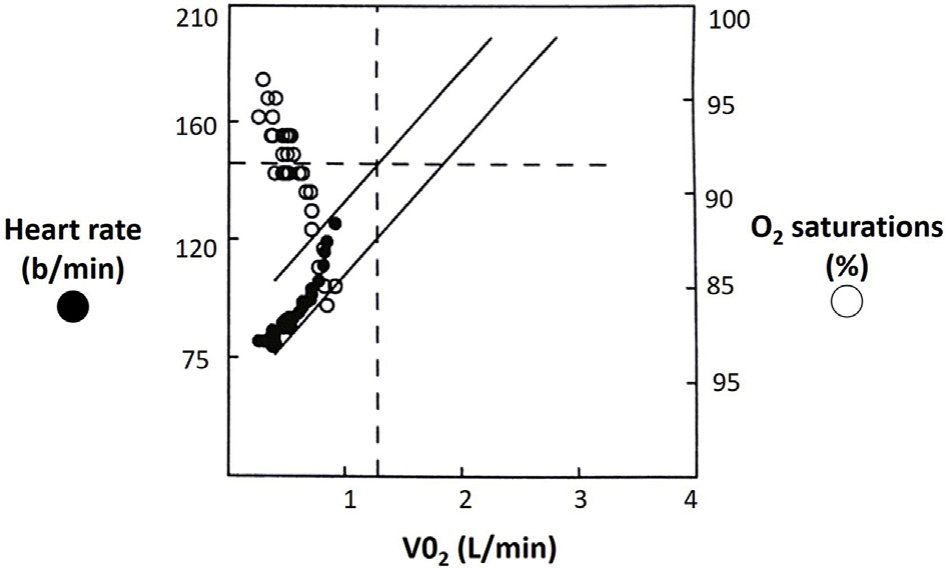
Cardiopulmonary exercise test showing relationship between oxygen saturation, heart rate and oxygen consumption (V0_2_) during exercise.

Staging positron emission tomography confirmed an avid lesion confined to the upper lobe of the left lung with no extrapulmonary disease. After an intensive 3 week home rehabilitation programme, respiratory function tests revealed a forced expiratory volume in 1 second (FEV1) of 0.72L (34% mean predicted; normal range > 1.55L), forced vital capacity (FVC) of 1.93L (69% predicted, normal reference range >2.13L), and a carbon monoxide transfer factor (TLCO), corrected for haemoglobin, of 10.0 ml/min/mmHg (normal range > 15.2 ml/min/mmHg). Oxygen saturation was 93% on room air. A cardiopulmonary exercise test (CPET) had demonstrated little ventilatory reserve with profound arterial desaturation to 84% on peak exercise (Fig. 1). However peak oxygen consumption was reassuringly normal measured at 16.7 ml/min/kg and the nadir minute ventilation/carbon dioxide (VE/VCO_2_) slope was of 24 implying a limited risk of perioperative cardiovascular morbidity [10,[12], [13], [14], [15]]. The estimated anaerobic threshold was 10.9 ml/kg/min. Oxygen pulse for the duration of exercise also appeared normal, but there was a substantial abnormality in minute ventilation/oxygen (VE/VO_2_) gradient suggesting a predominately respiratory cause of exercise limitation. Further risk stratification included a ventilation lung scan using Tc-99 m, followed by pulmonary perfusion scintigraphy. Quantitative evaluation of regional perfusion, tomographic imaging and low dose computed tomography further confirmed marginal regional ventilation and perfusion suggesting an inability to tolerate one lung anaesthesia (Table 1).

**Table 1:** Single photon emission computed tomography (SPECT) and computed tomography (CT) assessment of differential perfusion and ventilation using geometric means.

	Left upper Zone	Left mid zone	Left lower zone	Right upper zone	Right mid zone	Right lower zone	Summary
Differential pulmonary perfusion	18%	35%	10%	17%	21%	3%	The left and right lung contribute 63% and 37% respectively to total pulmonary perfusion
Differential pulmonary ventilation	17%	33%	11%	12%	24%	3%	The left and right lung contribute 61% and 39% respectively of total pulmonary ventilation

Assessment of differential lobar perfusion using SPECT/CT	Total right lung: total left lung = 35%: 65%			Contribution of left upper lobe to total lung perfusion = 38%			Contribution of left lower lobe to total lung perfusion = 27%
Assessment of differential lobar ventilation using SPECT/CT	Total right lung: total left lung = 34%: 66%			Contribution of left upper lobe to total lung ventilation = 33%			Contribution of left lower lobe to total lung ventilation = 33%

On the basis of these conflicting results it was uncertain if one-lung ventilation could be tolerated for lung resection surgery. The patient's respiratory dysfunction was marginal as evident by both the dynamic respiratory function tests (FEV1 of 0.72L and TLCO of 10.0 ml/min/mmHg) and the ventilation/perfusion imaging; conversely the cardiopulmonary exercise test was limited by ventilation, however reported some prognostic parameters, namely the peak oxygen consumption, within acceptable thresholds. Therefore, to further elucidate whether the patient would be able to tolerate definitive lung resection surgery, an elective oxygen challenge assessment was undertaken to gain a functional and real-time assessment of gas exchange during single lung ventilation.

**Fig. 2:**
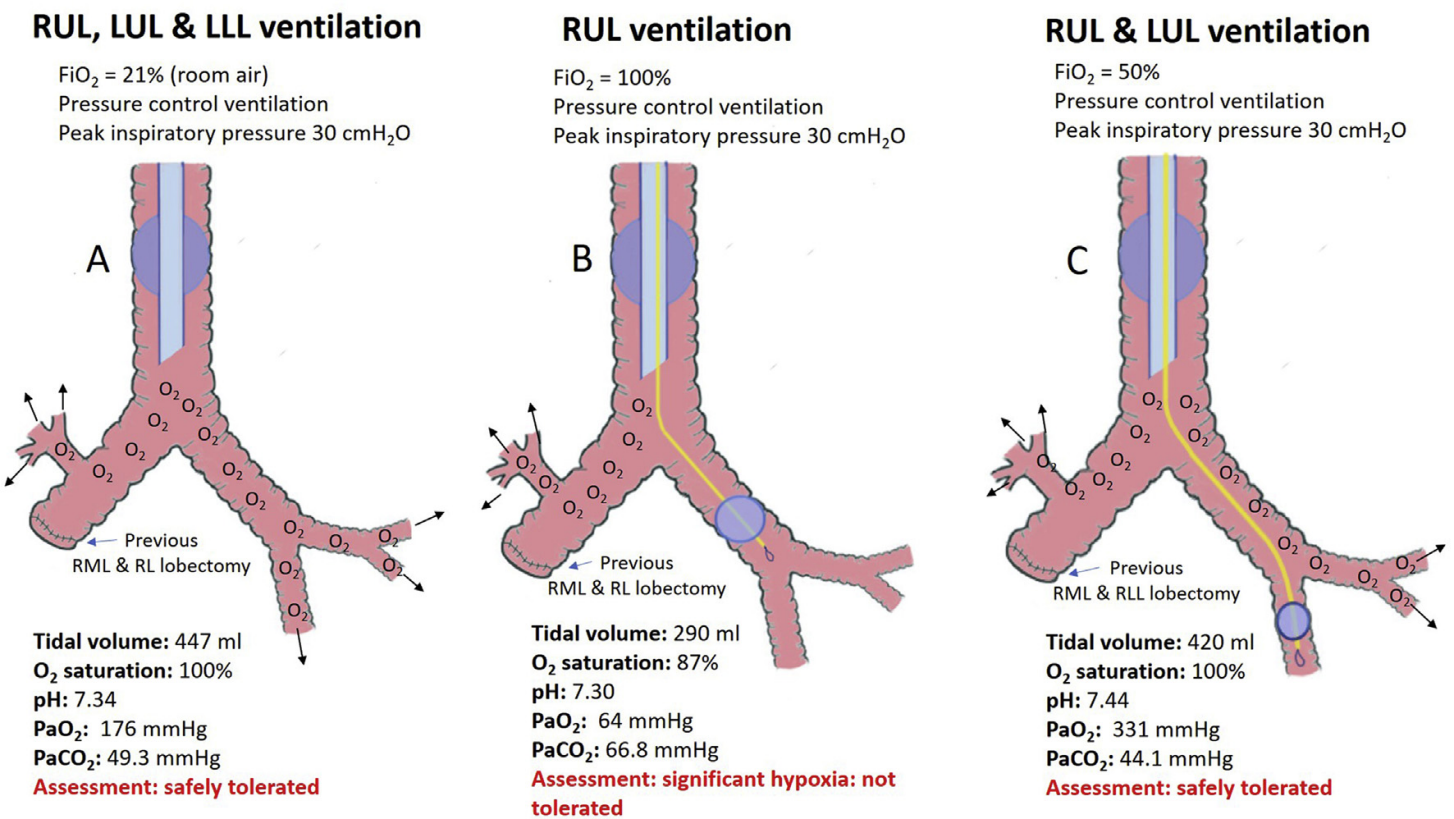
Intraoperative oxygen challenge test with positioning of the bronchial blocker and corresponding arterial blood gas measurements.

With patient consent, general anaesthesia was induced by the anaesthetist with the patient in the supine position. The patient's trachea was intubated using a 8.0 mm single lumen endotracheal tube. Anaesthesia was then maintained with sevoflurane and deep neuromuscular paralysis was provided with rocuronium. First, both lungs were ventilated, using pressure control mode of ventilation with 30 mmHg peak inspiratory pressure and 5 cmH_2_O positive end expiratory pressure. This achieved a tidal volume of approximately 6–7ml/kg. With the FiO_2_ set at 21%, adequate gas exchange was attained (Fig. 2A). With these ventilatory settings, the patient achieved 100% oxygen saturation and maintained a PaO_2_ of 176 mmHg. Then, a 7.0Fr/65cm Arndt™ endobronchial blocker (Cook Medical, IN, USA) with a spherical balloon was sited by the anaesthetist under fibreoptic guidance to the level of the left main bronchus. With the balloon inflated we occluded ventilation of the left lung, allowing isolated ventilation of the residual right upper lobe (Fig. 2B). The patient desaturated profoundly, and responded poorly to increased inspired oxygen, remaining significantly hypoxic (PaO_2_ = 64 mmHg) with an FiO_2_ of 100% being delivered. It was concluded that the patient would not tolerate definitive lung resection surgery under these conditions. The bronchial blocker was then deflated, carefully re-advanced under fibreoptic guidance into the left lower lobe bronchus, then reinflated allowing assessment of ventilation of both the residual right lung, left upper lobe including the lingual segments (Fig. 2C). The patient was able to maintain an oxygen saturation of 100% on an FiO_2_ of 21% with a PaO_2_ of 167 mmHg and was deemed safely able to tolerate anaesthesia and lung separation. This oxygen challenge also confirmed the safest lung separation device (i.e. use of a bronchial blocker, in contrast to a conventional double lumen tube). There were no adverse events and the procedure was well tolerated without any complication.

Post intervention, after further multidisciplinary discussions exploring the patient's values and preferences about the benefits and risk of elective left lower lobe superior segmentectomy and non-surgical alternatives, the patient decided to proceed with stereotactic ablative radiotherapy (SABR).

## 3 Discussion

Whilst CPET remains an important and objective test of assessing functional capacity of patients undergoing high risk surgery, its prognostic value in patients with severe COPD who have undergone previous lung resection surgery is uncertain. In this case, the patient's exercise capacity, as reflected by the peak VO_2_ was reassuringly normal. The VE/CO_2_ slope was also suggestive of a low perioperative risk [9,[12], [13], [14]]. Given the patient's history of severe COPD, previous lung resection surgery, and severe derangements in differential lung perfusion and ventilation testing, this intraoperative oxygen challenge test was an effective intervention to definitely assess safety and tolerance of single lung function under general anaesthesia and provide an informed decision for the patient about the appropriate therapeutic options.

Lung resection surgery remains the cornerstone of curative therapy for the majority of lung cancers. A myriad of evidence-based clinical practice guidelines and consensus statements from the USA [1,2], Europe [3,4] and Australia [5], advocate surgery as the standard treatment of stage I-II non-small lung cancer in patients with adequate pulmonary reserve to withstand lobectomy. This treatment confers the highest chance of local control and 5-year survival when compared to alternative treatments. When lobectomy may not be able to be safely performed due to limited pulmonary reserve, as in our case, many of the benefits of lobectomy are maintained by anatomic sub-lobar resection/segmentectomy and mediastinal lymph node clearance [15]. These benefits include resection of intra parenchymal, hilar and mediastinal lymph node basin, complete staging, and comprehensive histological, molecular and immune assessment. These in turn inform prognosis and guide adjuvant or palliative treatments. At present lung cancer treatment guidelines [[1], [2], [3], [4], [5]] advocate sub-lobar anatomic resection in patients unfit for lobectomy. For patients with stage I-II non-small lung cancer with limited pulmonary reserve, endobronchial ablative measures are currently at best considered exploratory and should only be employed in the context of an ethically approved clinical trial [3]. We did not offer endobronchial ablative therapy to our patient given her suitability to SABR and the superior body of evidence with respect to SABR safety, efficacy and medium-term survival outcomes [16]. Irrespective of treatment options, it's paramount to adopt an individualised approach and engage patients and their families in joint shared decision-making processes predicated upon effective communication of risk [17].

Our institution has been at the forefront of preoperative risk stratification for patients with lung cancer undergoing lung resection, reporting methods such as the predicted postoperative product as a predictor of surgical mortality [18]. We have previously reported perioperative risk stratification in various populations including after induction therapy [19] and using advanced molecular imaging technologies [20]. In the present case, we further attempt to more accurately stratify an individual patient's risk in order to clearly communicate and engage in informed and shared decision making.

## 4 Conclusions

In patients with severe COPD, traditional physiological parameters obtained during CPET and pulmonary function tests may be insufficient to provide an informed decision to the treating clinicians and their patients about appropriate perioperative risk. The intraoperative oxygen challenge test is another risk stratification tool to assist in assessment of safety and tolerance of anaesthesia for patients being considered for lung resection. This test also allowed the multidisciplinary managing teams to better counsel this patient who was initially very committed to further surgery, however based on the information provided by our method of assessment of functional pulmonary reserve, finally chose a non-surgical treatment option.

## Ethical approval

The Human Research Ethics Committee at Austin Health requires written informed consent for the publication of case reports. Written informed consent from the patient was obtained for publication of this case report and accompanying images.

## Sources of funding

This research did not receive any specific grant from funding agencies in the public, commercial, or not-for-profit sectors.

## Authors' contributions

**Luka Cosic, Maleck Louis**: conceptualisation, literature review, writing of manuscript – original draft, visualisation; **Patryck Lloyd-Donald, Lachlan F Miles**: literature review, data acquisition, writing of manuscript – original draft; **Tom Garry**: preparation of images; writing of manuscript – review and editing; **Stephen Barnett**: principle surgeon managing the patient, writing of manuscript – review and editing; **Laurence Weinberg**: conceptualisation, literature review, principle anaesthetist caring for patient, writing of manuscript – review and editing, coordinating author.

## Research registration number

1. Name of the registry: Not applicable
2. Unique Identifying number or registration ID: Not applicable

Hyperlink to the registration (must be publicly accessible): Not applicable.

## Guarantor

A/Prof Laurence Weinberg.

Director of Anaesthesia, Austin Hospital.

145 Studley Road, Heidelberg, Victoria, 3084, Australia.

laurence.weinberg@austin.org.au.

Phone: +613 9496 5000.

## Consent

Written informed consent was obtained from the patient for publication of this case report and accompanying images. A copy of the written consent is available for review by the Editor-in-Chief of this journal on request.

## Availability of data and materials

The cardiopulmonary exercise test, respiratory function tests and quantitative evaluation of regional perfusion, tomographic imaging and low dose computed tomography are available from the corresponding author on reasonable request.

## Declaration of competing interest

The authors declare they have no conflicts of interests.

## Appendix A Supplementary data

SCARE Guideline Checklist_26_10_2019 SCARE Guideline Checklist_26_10_2019
